# Synthesis and Characterization of Dual Natural Quercetin/Fucoidan Gene Delivery Nanoplatform for Synthetic Lethality in BRCA-Deficient Tumors

**DOI:** 10.3390/polym18111314

**Published:** 2026-05-26

**Authors:** Jih-Hao Yeh, Shih-Yu Huang, Ching-Chun Chu, Chun-Tao Su, Hung-Wei Cheng, San-Yuan Chen

**Affiliations:** 1 Department of Materials Science and Engineering, National Yang Ming Chiao Tung University, Hsinchu 300093, Taiwan; aasd3431@gmail.com (J.-H.Y.); e881122e881122@gmail.com (S.-Y.H.); seaftseaft@gmail.com (C.-C.C.); chuntaosu.en09@nycu.edu.tw (C.-T.S.); 2 Department of Biological Science and Technology, China Medical University, Taichung 406040, Taiwan; 3 Graduate Institute of Biomedical Science, China Medical University, Taichung 406040, Taiwan; 4 School of Dentistry, College of Dental Medicine, Kaohsiung Medical University, Kaohsiung 807378, Taiwan

**Keywords:** quercetin, Poly(ADP-ribose) polymerase inhibition, synthetic lethality, nanomedicine, PD-L1 blockade, BRCA1/2-mutant tumors

## Abstract

Cancer is a complex and evolutionary disease, with the development of different types of cancers leading to various different defective gene mutations. Synthetic lethality is a genetic-level precision medical strategy. Currently, treating BRCA (BReast CAncer)-mutated breast or ovarian cancer cells with a chemical inhibitor (Poly(ADP-ribose) polymerase, PARPi) is a typical synthetic lethal application in clinical practice. However, PARPi therapy has been found to cause off-target effects and therapy-induced immune escape driven by PD-L1 upregulation, allowing for cancer cells to escape attack from the immune response. To overcome these challenges, we developed a core–shell structure comprising a hydrophobic core of quercetin (Q)-mediated PARP inhibition and iron oxide nanoparticles (IONPs), enveloped by a hydrophilic fucoidan (Fu) shell to encapsulate short hairpin RNA targeting Programmed Death Ligand 1 (shPD-L1) for efficient gene transfection (shPD-L1@QIO@Fu). Structurally, the incorporation of quercetin into the intermediate hydrophobic layer enables modulate of the PARP effect, while the inner aqueous core with shPD-L1 gene silencing can inhibit the expression of PD-L1 protein. In this study, we proved that shPD-L1@QIO@Fu demonstrated a dual therapeutic mechanism against BRCA-mutant cancer cells by inducing extensive DNA double-strand breaks and promoting apoptosis. Furthermore, the combined action of quercetin-mediated DNA damage and shPD-L1-driven PD-L1 suppression led to a significant reduction in PD-L1 mRNA to approximately 5% at 72 h and decreased surface PD-L1 below baseline by 96 h. This effectively suppresses PARPi-induced PD-L1 upregulation and enhances antitumor immunity. These findings demonstrate the therapeutic efficacy of shPD-L1@QIO@Fu nanomedicine, providing a promising foundation for advanced co-delivery strategies to synergize PARP inhibition mediated synthetic lethality with immune checkpoint blockade in next-generation precision medicine.

## 1. Introduction

Cancer is regarded as a complex disease with multiple genetic changes that result in excessive growth, metastasis, and drug resistance [1,2,3]. Unlike conventional targeted therapies, synthetic lethal therapies have been considered some of the most effective cancer treatments in the last decade. This approach enables indirect targeting of cancer mutations by identifying alternative synthetic lethal targets, ranging from oncogenes and tumor suppressors to DNA repair genes and cancer metabolism pathways. This selective mechanism also allows for the straightforward identification of patients based on their specific cancer cell genetic mutations [4]. Poly(ADP-ribose) polymerase (PARP) inhibitors (PARPi), which target the inhibition of particular DNA repair pathways, have been approved for clinical use to target BRCA1/2-mutated breast tumors, ovarian cancer and prostate cancer based on synthetic lethality [5]. With the development of new experimental and computational approaches, the U.S. Food and Drug Administration (FDA) has now approved four PARP inhibitors for clinical anti-cancer therapy such as Olaparib (Lynparza^®^) as a PARPi, representing a clinical translation of synthetic lethality by preventing the repair of single-strand DNA breaks (SSBs) [6]. This results in stalled replication forks and subsequent double-strand breaks (DSBs) in BRCA-mutant cells, which lack efficient homologous recombination (HR) repair [7,8]. However, despite the initial clinical success of PARP inhibitors (PARPi), their therapeutic efficacy is frequently limited by several critical challenges [9]. Notably, PARPi treatment has been shown to induce PD-L1 upregulation on tumor cells via activation of the glycogen synthase kinase-3β (GSK3β) signaling pathway, thereby allowing for cancer cells to evade the immune system [10]. Consequently, combination strategies integrating PARPi with immunotherapy—particularly immune checkpoint blockade targeting PD-L1—have demonstrated synergistic antitumor effects [11,12]. For instance, Ding et al. reported that co-administration of a programmed cell death protein 1 (PD-1) inhibitor with Olaparib significantly enhanced therapeutic efficacy by activating the stimulator of interferon genes (STING) pathway and augmenting PD-L1 expression in high-grade serous ovarian cancer [13]. Nevertheless, the FDA-approved PARPi Olaparib is hindered by suboptimal bioavailability and limited tumor accumulation [14]. Moreover, adaptive upregulation of endogenous PD-L1 mRNA expression remains a major mechanism of immune escape, contributing to the modest overall response rates observed in solid tumors, which typically remain below 30% [15,16,17].

Recently, the natural flavonoid quercetin has been widely studied as a potential alternative PARPi due to its accessibility, cost-effectiveness, and promising safety profile [18,19,20]. In addition, quercetin exhibits a broad spectrum of anti-cancer activities, including immunomodulation, inhibition of angiogenesis, and regulation of the tumor microenvironment [21,22,23,24,25]. However, the clinical application of quercetin is hindered by its poor aqueous solubility, low chemical stability, and rapid metabolic degradation [26,27]. To address these limitations, developing a nanoplatform to incorporate quercetin as a stable structural element not only overcomes physicochemical barriers but also serves as a foundation for effective PARPi delivery to achieve synthetic lethality by preventing the repair of single-strand DNA breaks (SSBs).

Nanostructured delivery platforms can substantially enhance the bioavailability and tumor accumulation of PARP inhibitors. However, effectively counteracting the adaptive PD-L1 upregulation triggered by PARP inhibition requires a therapeutic strategy capable of achieving sustained and robust PD-L1 suppression. Recent advances have highlighted the efficacy of multifunctional nanosystems that integrate small-molecule inhibitors with immunotherapeutic modalities, including immune checkpoint-targeting antibodies and RNA interference (RNAi)-based gene silencing agents. Such combinatorial nanomedicine approaches enable coordinated modulation of DNA damage responses and tumor immune evasion pathways, thereby offering a promising avenue to amplify therapeutic synergy and overcome resistance mechanisms associated with PARPi monotherapy [28,29,30]. Among these approaches, RNA interference (RNAi) technologies—including small interfering RNA (siRNA) and short hairpin RNA (shRNA)—provide a precise strategy for silencing PD-L1 mRNA expression, thereby restoring antitumor immune activity. Compared with siRNA, whose gene-silencing effect is inherently transient and may lead to suboptimal immune reactivation [31], shRNA enables continuous intracellular generation of siRNA, resulting in more prolonged and stable gene silencing [32,33,34,35]. Therefore, this sustained knockdown capability helps ensure effective inhibition of PD-L1 upregulation.

Building upon these insights, we developed a novel dual natural bioactive compound-based gene delivery nanomedicine, shPD-L1@QIO@Fu, which integrates quercetin (Q), fucoidan (Fu), superparamagnetic iron oxide nanoparticles (IONPs), polyethyleneimine (PEI), polyethylene glycol (PEG), and an shPD-L1 DNA plasmid. To enable efficient gene delivery, the shPD-L1 plasmid is electrostatically complexed with PEI, facilitating intracellular transport and effective PD-L1 gene silencing. Fucoidan (Fu), is a hydrophilic and highly sulfated natural product extracted from *Fucus vesiculosus* [36,37]. In addition, several studies have reported that fucoidan as an alternative medicine in animal and human clinical trials have proved that combining fucoidan with clinical therapeutic agents can alleviate the side effects of anti-cancer chemotherapy [38,39]. To synthesize the core–shell structure, double emulsion capsules (DECs) are usually prepared by a two-step emulsifying process, with surfactants often added in one or both steps to stabilize emulsions. Here, with the aim of obtaining the same core–shell construct from a surfactant-free one-step emulsifying process, a nanoplatform is rationally engineered by incorporating intrinsic PARPi hydrophobic quercetin with an antitumor hydrophilic fucoidan to construct the shell, which includes IONPs as DEC stabilizers [40,41]. Through this coordinated co-delivery strategy, the nanomedicine integrates quercetin-mediated PARP inhibition with RNA interference-driven PD-L1 suppression, thereby maximizing therapeutic efficacy against BRCA-mutant cancer cells. Notably, the design strategically exploits quercetin as both a functional structural component and a bioactive therapeutic agent, demonstrating the therapeutic potential of shPD-L1@QIO@Fu to achieve synergistic chemo/gene therapy in BRCA-mutant cancers.

## 2. Experimental

### 2.1. Materials

Tris(acetylacetonato)iron (III), 1,2-hexadecanediol (97%), oleic acid (90%), oleylamine (>70%), benzyl ether (99%), ethyl acetate, fucoidan (Mw = 15,000–25,000), poly(ethylene glycol) (PEG) (Mn = 3350), polyethylenimine (MW = 25,000), Folin & Ciocalteu’s phenol reagent, paraformaldehyde, dimethyl sulfoxide (DMSO), and Fluoroshield with DAPI histology mounting medium were purchased from Sigma-Aldrich (St. Louis, MO, USA). Quercetin dihydrate (97%) was purchased from Alfa Aesar (Ward Hill, MA, USA). Fetal bovine serum (FBS), trypsin-EDTA, Dulbecco’s Modified Eagle Medium (DMEM), Qdot^TM^ 545 ITK^TM^ Organic Quantum Dots, Alexa Fluor^®^ 488 Phalloidin, Dead Cell Apoptosis Kit with Annexin V Alexa Fluor^TM^ 488 & Propidium Iodide (PI), and PE Anti-Mouse CD274/PD-L1 antibody were purchased from Thermo Fisher Scientific (Waltham, MA, USA). Triton-X 100 was purchased from J.T. Baker (Phillipsburg, NJ, USA). Phospho-Histone H2A.X (Ser139) (clone 20E3) Rabbit mAb was purchased from Cell Signaling Technology (Danvers, MA, USA). Fluorescence mounting medium was obtained from Dako Agilent Technologies (Santa Clara, CA, USA). Phosphate buffered saline (PBS) was purchased from AMRESCO (Solon, OH, USA). Cell counting Kit-8 was purchased from Dojindo (Kumamoto, Japan). Purified Rat Anti-Mouse CD16/CD32 (Mouse BD Fc Block) was obtained from BD Biosciences (San Jose, CA, USA). Recombinant Anti-M6PR (cation independent) antibody and Goat Anti-Rabbit IgG H&L (Alexa Fluor 488) were purchased from Abcam (Cambridge, UK). TOOLSmart RNA Extractor was purchased from Toolsbiotech (New Taipei City, Taiwan). GelRed Nucleic Acid Stain was purchased from Biotium (Fremont, CA, USA). pGPU6/GFP/Neo-cd274-mus-890 shRNA expression pDNA was obtained from Genepharma (Shanghai, China). 4T1 BRCA gene mutated-cells were generated via genetic editing by Academia Sinica (Taipei, Taiwan). CdSe/ZnS core/shell quantum dots (QDs) were purchased from Ocean NanoTech (San Diego, CA, USA).

### 2.2. Synthesis of Superparamagnetic Iron Oxide Nanoparticles (SPIOs)

In this study, iron oxide nanoparticles (IONPs) were synthesized following a modified procedure based on the thermal decomposition method reported by Sun et al. [42]. Iron(III) acetylacetonate (Fe(acac)_3_; 1.059 g, 2 mmol), 1,2-hexadecanediol (3.87 g, 10 mmol), oleylamine (0.9675 mL, 6 mmol), oleic acid (0.9675 mL, 6 mmol), and benzyl ether (30 mL) were mixed in a three-necked flask under an inert nitrogen atmosphere. The reaction mixture was subjected to a sequential heating process: initially held at 100 °C for 1 h, subsequently heated to 200 °C for 1 h, and finally maintained at 285 °C for 30 min. After the reaction was completed, the mixture was cooled to room temperature. It was then centrifuged at 4000 *rcf* for 10 min to remove the supernatant, the product re-dissolved in alcohol, with centrifugation repeated at 4000 *rcf* for 10 min to purify the product 3 times.

### 2.3. Preparation of Quercetin/Fucoidan Magnetic Nanoparticles (shPD-L1@QIO@Fu, QIO@Fu)

The shPD-L1@QIO@Fu and QIO@Fu nanoparticles were synthesized using a modified water-in-oil-in-water (*W*/*O*/*W*) double emulsion method. First, to prepare the primary water-in-oil (*W*/*O*) emulsion, polyethylenimine (PEI, 240 µL of 0.27 µg/µL solution) was added the ethyl acetate (600 µL) consisting of quercetin (0.75% *w*/*v*) and iron oxide nanoparticles (IONPs, 1.5 mg) emulsified at 1 W and 70% cycle for 25 s using a homogenizer (UP200Ht, ultrasonic processor, Hielscher). Next, the resulting *W*/*O* emulsion was thoroughly mixed with the outer aqueous phase. Based on preliminary studies that evaluated fucoidan volumes of 1.8, 2.0, 2.2, and 2.4 mL, a volume of 2.2 mL of fucoidan solution (5 mg/mL) was selected. This mixture then underwent secondary emulsification at 10 W and 70% cycle for 1 min and 10 s to form the *W*/*O*/*W* emulsion.

Subsequently, the organic solvent, ethyl acetate, was removed by rotary evaporation at 55 °C. The synthesized nanoparticles were then purified using a magnetic separation device (MagniSort (eBioscience, San Diego, CA, USA)) and thoroughly washed four times with deionized water.

For optimal stability and morphology, concentrations of 0.5, 1.0, 1.5, and 2.0 mg/mL polyethylene glycol (PEG) were added to the fucoidan (5 mg/mL). A concentration of 2 mg/mL PEG was determined. For the preparation of shPD-L1@QIO@Fu nanoparticles, shPD-L1 (50 µg, with an N/P ratio = 3) was combined with the PEI in the initial hydrophilic phase and followed the identical protocol.

### 2.4. Characterization of shPD-L1@QIO@Fu, QIO@Fu

The particle size and zeta potential of the shPD-L1@QIO@Fu, QIO@Fu were characterized by dynamic light scattering (Delsa^TM^ Nano C particle analyzer, Beckman Coulter, Brea, CA, USA). The morphology of the nanoparticles was determined by scanning electron microscope (SEM, JEOL-6700, JEOL, Tokyo, Japan) and transmission electron microscope (TEM, JEM-2100, JEOL, Tokyo, Japan). The equivalent quercetin content in shPD-L1@QIO@Fu, QIO@Fu was measured by Folin–Ciocalteu colorimetry [43]. The DNA loading efficiency of shPD-L1 was measured by GelRed staining, and the DNA content in the supernatant was extrapolated by Fluorescence Spectrophotometer (F-7000, Hitachi, Tokyo, Japan) measurement of equally reduced PEI and DNA plasmid absorbance at 600 nm.

### 2.5. N/P Ratio

The N/P ratio can be described as the molar ratio of ionizable amine nitrogen atoms (N) present in the cationic polymer (polyethyleneimine, PEI) to the phosphate groups (P) within the nucleic acid (DNA) backbone. This ratio represents a critical physicochemical parameter that significantly influences the formation, stability, and transfection efficiency of the resulting polymer/DNA complexes, particularly those designed as gene delivery vectors. Polyethyleneimine (PEI) is a molecule containing four primary amines, with an average 11 molecular weight of 473 in each monomer. DNA, on the other hand, contains a phosphate group, and its average molecular weight is approximately 325. Therefore, the N/P ratio was calculated as follows (Equation (1)):

(1) $$N/P=2.748\times \frac{mPEI}{mDNA}$$

where mPEI is the mass of PEI in μg and mDNA is the mass of DNA in μg in each sample. The constant (2.748) in the formula was calculated as the molecular weight of DNA (325) divided by the molecular weight of a single primary amine (473/4). The calculation represents an approximation and may differ from conventional methods based on repeating unit molecular weights of PEI.

### 2.6. PEI-DNA Plasmid Binding Ability

In order to evaluate the ability of PEI to carry DNA, we measured the binding ability of PEI-DNA under different N/P ratios through potential changes. Varying amounts of PEI solution were added to a fixed quantity (50 μg) of plasmid DNA in individual tubes to achieve target N/P ratios (0, 2, 4, 6, 8). The zeta potential change was measured by dynamic light scattering, DLS, and electrophoretic light scattering, ELS (Delsa^TM^ Nano C particle analyzer, Beckman Coulter, Brea, CA, USA).

### 2.7. Cell Culture

4T1/4T1 BRCA-mutated breast cancer cells were maintained in DMEM supplemented with 10% Fetal Bovine Serum (FBS) and 1% penicillin–streptomycin–neomycin (PSN) solution in 75T culture flasks at 37 °C under 5% CO_2_.

### 2.8. Transfection Efficiency of Different N/P Ratio in 4T1 Cells

4T1cells incubated in DMEM with 10% FBS in a 24-well plate at a density of 2 × 10^4^ cells per well and cultured for 24 h. The following day, we added a different N/P ratio (2, 3, 4, 6) of PEI/DNA plasmid in no FBS DMEM culture medium. After 24 h, it was supplemented with 10% FBS, and observation continued at 48 h with a fluorescence microscope (Eclipse TE2000-U, Nikon, Tokyo, Japan).

### 2.9. Cellular Uptake and Subcellular Localization of shPD-L1@QIO@Fu

To assess the cell endocytosis behavior, 4T1 cells were incubated with shPD-L1@QIO@Fu. To visualize and quantify the cellular uptake by fluorescence microscopy and flow cytometry, hydrophobic CdSe/ZnS core/shell quantum dots (QDs, 620 nm) were added to organic solvents and then encapsulated into shPD-L1@QIO@Fu according to the above emulsion process.

To observe cancer cell uptake, 1 × 10^5^ 4T1 cells per well were incubated on microscope slides for 24 h. Then, the cells were incubated with shPD-L1@QIO@Fu for 1, 4, 8, and 24 h. After incubation, the cells were stained with nuclei (blue) and cytoskeleton (green) by DAPI (excitation at 405 nm, emission at 430 to 480 nm in the blue channel) and Alexa Fluor^®^ 488 Phalloidin (excitation at 488 nm, emission at 500 to 550 nm in the green channel). The QD-labeled nanoplatforms were tracked in the red channel (excitation at 561 nm, emission at 600 to 640 nm). The observation of the subcellular localization of shPD-L1@QIO@Fu was conducted using a fluorescence microscope (Eclipse TE2000-U, Nikon, Tokyo, Japan) and Multiphoton and Confocal Microscope System: TCS-SP5-X AOBS (Leica, Germany). To quantify the uptake of QD-labelled shPD-L1@QIO@Fu, 4T1 cells were incubated with shPD-L1@QIO@Fu for 1, 4, 8, and 24 h. Next, cells were harvested with trypsin, and the fluorescence intensity was measured in BL3 channel by flow cytometer (Novocyte Flow Cytometer, ACEA Biosciences, San Diego, CA, USA).

### 2.10. Intracellular Lysosomal Uptake Pathway

4T1 cells were maintained in DMEM supplemented with 10% FBS in 6-well plates at a density of 1 × 10^5^ cells per well and cultured for 24 h. The next day, the added QD-labelled shPD-L1@QIO@Fu were incubated with cells in no FBS DMEM culture medium for 4, 16 h. After incubation, the cells were washed with PBS, fixed with 4% paraformaldehyde in PBS for 15 min, permeabilized with 0.25% Triton X-100 in PBS for 15 min, and then blocked with 4% FBS in PBS for 30 min. Cells were then stained overnight with recombinant anti-M6PR (cation-independent) antibody, Goat Anti-Rabbit IgG H&L (Alexa Fluor^®^ 488, excitation at 488 nm, emission at 500 to 550 nm in the green channel) for 1 h and DAPI (excitation at 405 nm, emission at 430 to 480 nm in the blue channel) fluorescence mounting medium. The intracellular localization and uptake of the QD-labelled constructs (excitation at 561 nm, emission at 600 to 640 nm) were visualized using a Leica TCS SP5-X AOBS confocal laser scanning microscope (Leica Microsystems, Wetzlar, Germany).

### 2.11. Cell Viability Assay In Vitro

To evaluate the cell viability assay and cytotoxicity assay, both 4T1 and 4T1 BRCA-mutated cells (5 × 10^4^) were maintained in DMEM supplemented with 10% FBS in 24-well plates. The following day, we added different concentrations of QIO@Fu (25, 50, 100, 200 μm, Quercetin) and incubated them with cells for 24 h with no FBS medium. Cell viability was determined using the CCK-8 method according to the manufacturer’s protocol.

### 2.12. Immunofluorescence Analysis of γ-H2AX Foci

4T1/4T1 BRCA-mutated cells were incubated in DMEM with 10% FBS in 6-well plates at a density of 1 × 10^5^ cells per well and cultured for 24 h. Following this initial culture, the cells were treated for 72 h with QIO@Fu, shPD-L1@QIO@Fu, or quercetin in fresh culture medium. After incubation, the cells were washed with PBS, fixed with 4% paraformaldehyde in PBS for 15 min, permeabilized with 0.25% Triton X-100 in PBS for 15 min, and then blocked with 4% FBS in PBS for 30 min at room temperature. The fixed cells were stained with Phospho-Histone H2A.X (Ser139) overnight, Goat Anti-Rabbit IgG H&L (Alexa Fluor^®^ 488, excitation at 488 nm, emission at 500 to 550 nm in the green channel) for 1 h, and DAPI (excitation at 405 nm, emission at 430 to 480 nm in the blue channel) fluorescence mounting medium. After cells are stained, they can be observed with a fluorescence microscope (Eclipse TE2000-U, Nikon, Tokyo, Japan).

### 2.13. Apoptosis Assays

4T1 BRCA-mutated cells were incubated in DMEM with 10% FBS at a density of 2 × 10^5^ cells per well and cultured for 24 h. The cells were incubated with quercetin and QIO@Fu. The cells treated with quercetin alone were used as control. The cells were harvested with trypsin and centrifugal purification (200 *rcf*, 5 min) in 150 mL 1×Annexin binding buffer after 72 h incubation, followed by double staining with Annexin V Alexa Fluor^TM^ 488 (6 mL) and propidium iodide (PI) (1 mL, 70 mg/mL) (Dead Cell Apoptosis Kit with Annexin V Alexa Fluor^TM^ 488 & Propidium Iodide (PI)). After 30 min incubation, samples were diluted with 500 mL 1× Annexin binding buffer and analyzed on a FACS Flow Cytometer (Novocyte Flow Cytometer, ACEA Biosciences, San Diego, CA, USA).

### 2.14. Transfection Observation of shPD-L1@QIO@Fu

1 × 10^5^ 4T1/4T1 BRCA-mutated cells were incubated in DMEM with 10% FBS in 6-well plates for 24 h. The cells were incubated with shPD-L1@QIO@Fu for 48 h. The experiment was observed with a fluorescence microscope (Eclipse TE2000-U, Nikon).

### 2.15. PD-L1 Expression in 4T1 BRCA-Mutated Cells

4T1/4T1 BRCA-mutated cells were incubated in DMEM with 10% FBS in 6-well plates at a density of 1 × 10^5^ cells per well and cultured for 24 h. The following day, we added shPD-L1@QIO@Fu, QIO@Fu, or quercetin, incubated with 4T1 BRCA-mutated cells, respectively, for 48, 72, and 96 h. The cells were harvested by trypsin EDTA and centrifugal purification (400 *rcf*, 5 min) in 100 mL blocking buffer (1% BSA in PBS solution), and we added Anti-Mouse CD16/CD32 (Mouse BD Fc Block) to block the Fc receptor (4 °C for 15 min). After blocking, samples were incubated with PE Anti-Mouse CD274/PD-L1 Antibody (4 °C for 1 h), and the fluorescence was assayed in a BL2 channel with flow cytometry (Novocyte Flow Cytometer, ACEA Biosciences, San Diego, CA, USA).

### 2.16. Quantitative Real-Time PCR Assay for PD-L1

4T1 BRCA-mutated cells were seeded at a density of 1 × 10^6^ cells into T75 flasks and cultured for 48 h. The culture medium was replaced with fresh medium on the evening prior to treatment. Subsequently, cells were treated for 72 h with predetermined concentrations of shPD-L1@QIO@Fu, QIO@Fu, or quercetin. Following treatment, cells were harvested using trypsin-EDTA (0.25%) and collected by centrifugation at 400 *rcf* for 5 min at 4 °C. Total RNA was isolated from the cell pellets using 1 mL of a suitable RNA extraction reagent. The purified RNA samples were quantified, and their integrity was assessed prior to storage at −80 °C. Primers for *PD-L1* and *Gapdh* were designed using the IDT PrimerQuest^TM^ Tool and analyzed with the OligoAnalyzer^TM^ Tool (Integrated DNA Technologies, Coralville, IA, USA). The primer sequences utilized were as follows:

*mPD-L1*(Forward): 5′-TGC AAC ACA TCC TCC ACA GAA C-3′;
*mPD-L1*(Reverse): 5′-AGG ACC GTG GAC ACT ACA ATG A-3′;
*mGAPDH*(Forward): 5′-CAT CAC TGC CAC CCA GAA GAC TG-3′;
*mGAPDH*(Reverse): 5′-ATG CCA GTG AGC TTC CCG TTC AG-3′.

Two micrograms (2 μg) of total RNA per sample were reverse transcribed into complementary DNA (cDNA) using the ToolsQuant II Fast RT Kit (Cat. No. KRT-BA06-2, BIOTOOLS, Taipei, Taiwan) according to the manufacturer’s instructions. The synthesized cDNA was subsequently diluted to a working concentration of 10 ng/µL and stored at −80 °C until use. Real-time PCR was performed using TOOLS 2xSYBR qPCR Mix (Cat. No. FPT-BB05, BIOTOOLS, Taipei, Taiwan), which contains SYBR Green I, in a 10 mL total reaction volume. Each sample was loaded in triplicate onto a 96-well PCR plate, and a no-template control (NTC) was included for each primer set. The qRT-PCR reactions were conducted on a CFX Connect Real-Time PCR Detection System (Bio-Rad Laboratories, Hercules, CA, USA). Briefly, the PCR conditions are summarized in Table 1.

### 2.17. Statistical Analysis

Data are expressed as mean ± standard deviation (SD) unless otherwise noted. Comparisons between two groups were performed using an unpaired two-tailed *t*-test. Statistical significance was set at *p*-value < 0.05.

## 3. Results and Discussion

### 3.1. Characterization of QIO@Fu

**Scheme 1:**
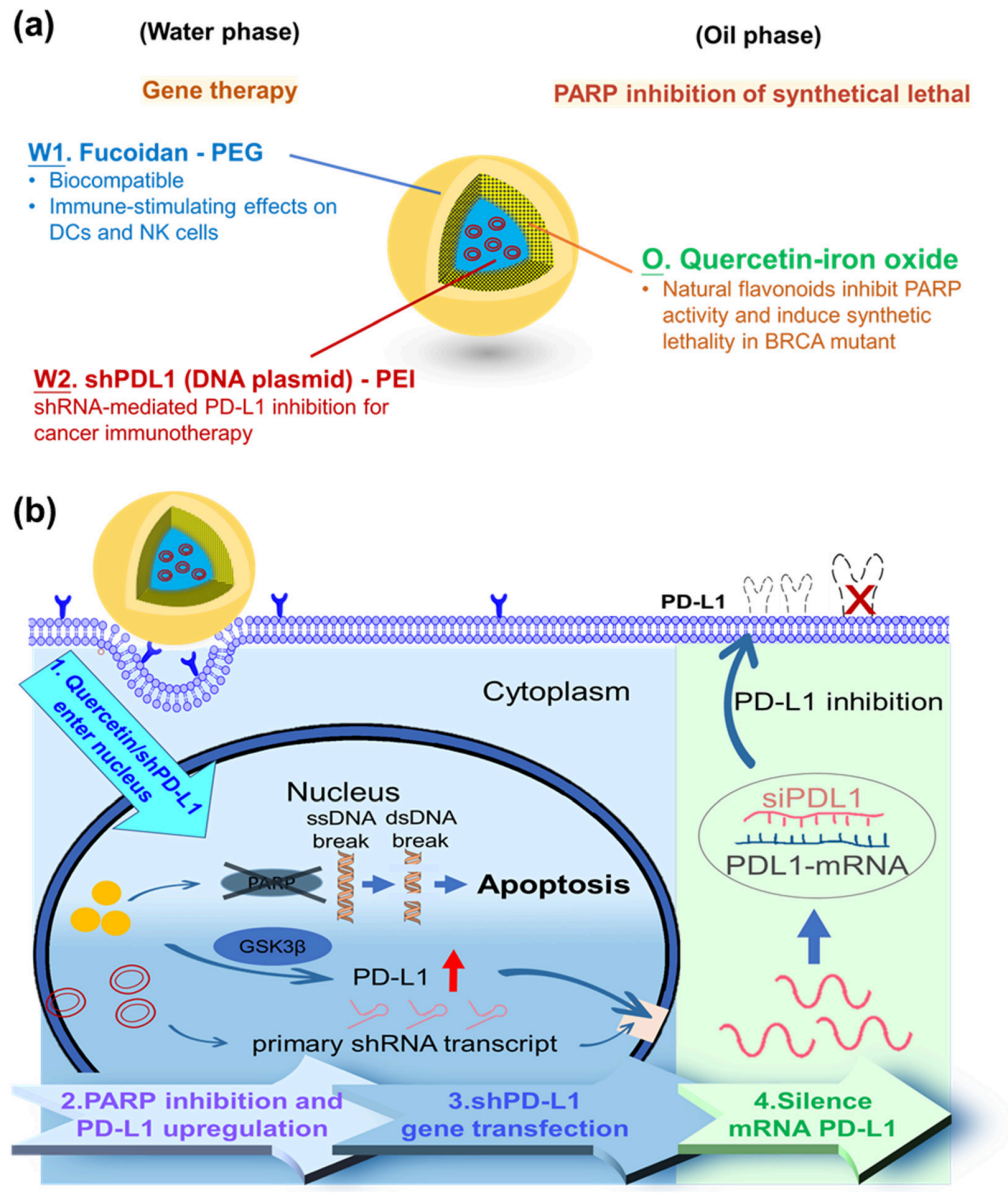
(**a**) Schematic illustration of the nanomedicine carried shPD-L1 DNA plasmid (shPD-L1@QIO@Fu) for synthetical lethal synergistic immunotherapy. (**b**) The proposed mechanism by which shPD-L1@QIO@Fu co-delivers a PARP inhibitor and shPD-L1 plasmid DNA to treat BRCA-mutant tumor cells.

Synthetic lethality is effective for cancers with DNA damage repair defects, but the upregulation of mRNA PD-L1 expression can limit its long-term efficacy. To address this, we developed shPD-L1@QIO@Fu, a nanomedicine engineered from the intrinsically therapeutic natural materials of fucoidan (Fu) and quercetin (Q) superparamagnetic iron oxide (IO), integrated with an shPD-L1 DNA plasmid in Scheme 1a. The middle shell layer of quercetin as PARP inhibitor and SPIO with magnetic navigation function can induce the synthetic lethality to promote immunity and enhance the accumulation of nanomedicine in tumor sites. The hydrophilic outermost layer uses the amphiphilic fucoidan to improve the biocompatibility of the nanomedicine and promote the activation of natural killer cells (NKs). The nanomedicine is designed to simultaneously inhibit PARP protein activity and silence PD-L1 expression, effectively tackling the issue of PD-L1 upregulation post-PARPi treatment. The treatment strategy of combining synthetic lethal therapy and immunotherapy is illustrated in Scheme 1b. After following cellular internalization, shPD-L1@QIO@Fu initiates a coordinated therapeutic process in BRCA-mutant triple-negative breast cancer (TNBC). Mechanistically, quercetin inhibits PARP, shutting down single-strand break repair and leading to the accumulation of DNA double-strand breaks (DSBs), which selectively kill BRCA-mutant (homologous recombination deficiency, HRD) tumor cells. In parallel, the transcribed shPD-L1 plasmid suppresses the DNA damage-induced PD-L1 upregulation, thereby preventing potential immune escape.

**Figure 1:**
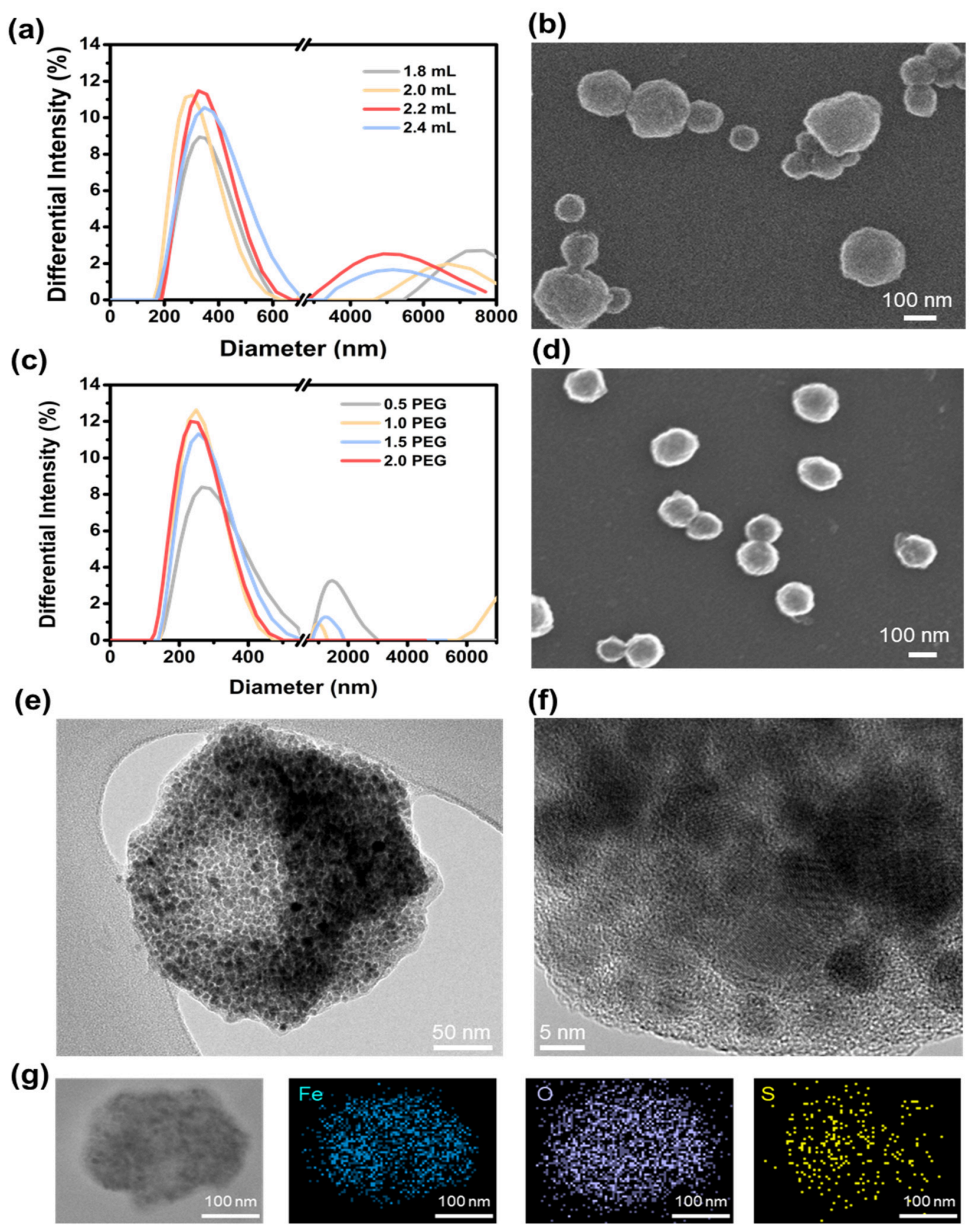
(**a**) Average particle size distribution of QIO@Fu synthesized at different W2 fucoidan solution volumes. (**b**) SEM image of QIO@Fu (W2 2.2 mL). Scale bar: 100 nm. (**c**) Size distribution of QIO@Fu at different PEG/fucoidan ratios. (**d**) SEM image of QIO@Fu (PEG ratio 2.0). Scale bar: 100 nm. (**e**) TEM image of QIO@Fu (PEG ratio 2.0). Scale bar: 50 nm. (**f**) High-resolution TEM showing IONPs within the shell of QIO@Fu. Scale bar: 5 nm. (**g**) TEM image and elemental mapping of iron (Fe), oxygen (O), and sulfur (S) for QIO@Fu acquired by TEM-EDS. Scale bars: 100 nm.

To evaluate the formation of the structural quercetin (Q)-based nanoparticles, QIO@Fu nanoparticles were initially synthesized using a *W*/*O*/*W* double-emulsion technique without the shPD-L1 plasmid. The QIO@Fu was designed to establish a well-defined core–shell structure, where the intermediate oil phase acts as a structural matrix for quercetin and iron oxide nanoparticles (IONPs), while creating an inner aqueous cavity for subsequent plasmid encapsulation. To optimize the colloidal stability, the oil-to-water ratio was adjusted by varying the volume of fucoidan in the outer aqueous phase (1.8, 2.0, 2.2, and 2.4 mL) and the corresponding particle size distributions were analyzed using dynamic light scattering (DLS). As shown in Figure 1a, QIO@Fu prepared using 2.2 mL of fucoidan exhibited the narrowest size distribution. This observation was supported by the polydispersity index (PDI), with 2.2 mL fucoidan (PDI = 0.199 ± 0.0145) yielding a narrower distribution compared to 2.4 mL fucoidan (PDI = 0.208 ± 0.031). Therefore, 2.2 mL fucoidan was selected as the optimal volume for subsequent experiments. However, a minor fraction of larger aggregates (5000 ± 1110 nm) persisted, with partial nanoparticle aggregation evident in the SEM image (Figure 1b) [44]. To further refine the nanoparticle morphology and suppress droplet coalescence, poly(ethylene glycol) (PEG) was incorporated as a steric stabilizer, thereby improving uniformity [45]. As evidenced in Figure 1c, with the addition of PEG at a PEG/fucoidan ratio of 2, the average particle size of QIO@Fu was significantly reduced to 250.8 ± 64.9 nm with no observed aggregation. In addition, the scanning electron microscopy (SEM) image in Figure 1d reveals that these nanoparticles were well-dispersed, exhibiting a uniform spherical shape. The transmission electron microscopy (TEM) image in Figure 1e further highlights the core–shell structure where both hydrophobic quercetin and iron oxide nanoparticles are localized within the inner shell surrounded by the outer fucoidan-PEG layer. An enlarged TEM image of clear black dots show that IO was within the shell of hydrophobic quercetin/hydrophilic fucoidan QIO@Fu in Figure 1f.

Elemental mapping by energy-dispersive X-ray spectroscopy (EDS) (Figure 1g) showed Fe signals predominantly in the shell; O distributed throughout the structure, indicative of the presence of oxygen from both IONPs and fucoidan; and a and S concentrated on the particle periphery. Therefore, the morphological and elemental analyses confirm the successful formation of the QIO@Fu core–shell structure with a fucoidan-coated surface.

**Figure 2:**
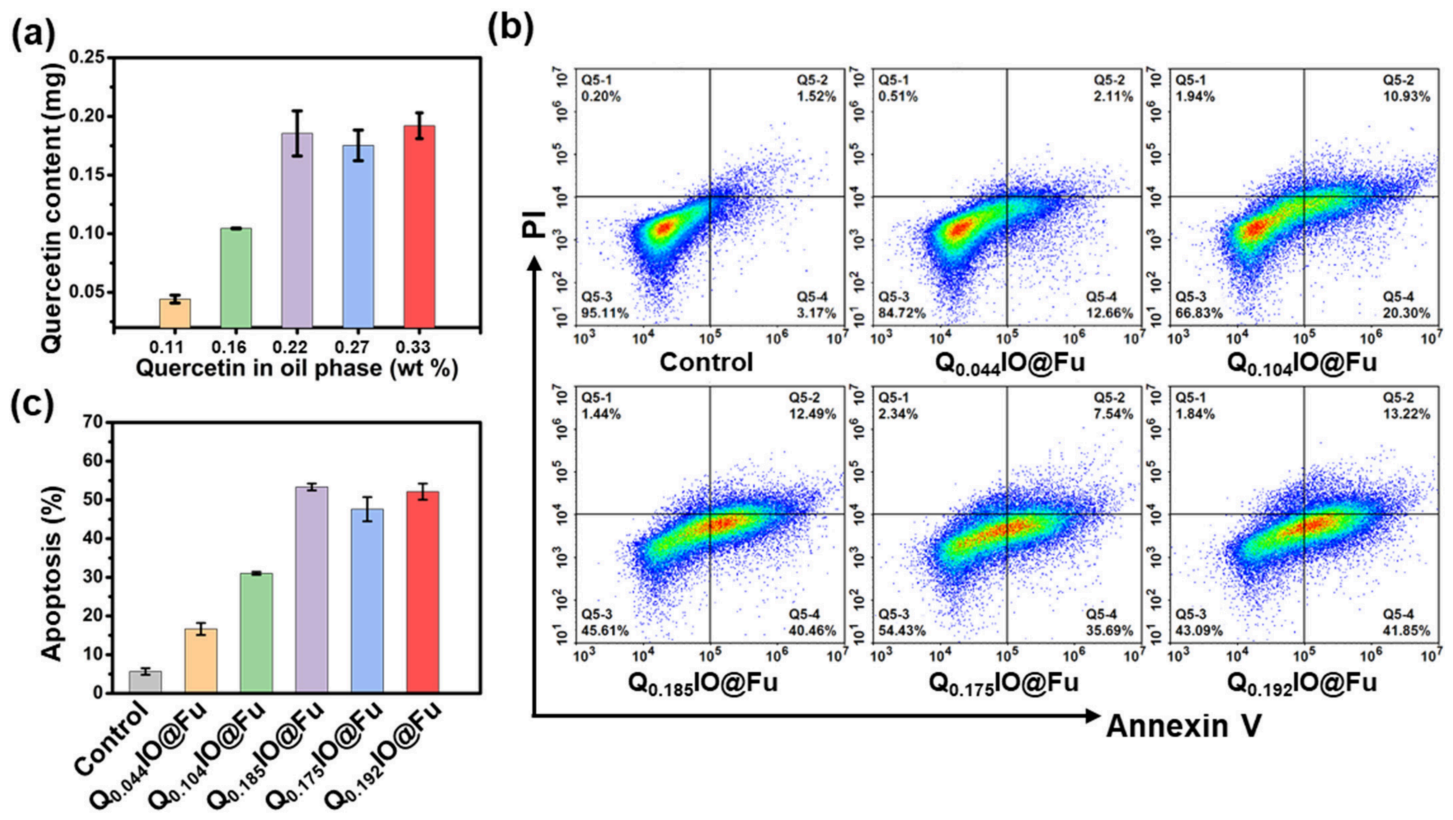
(**a**) Loading content of quercetin in QIO@Fu nanoparticles varied with the quercetin amount added in the oil phase. (**b**) Flow cytometry analysis of 4T1 BRCA-mutated cells after incubation with QIO@Fu nanoparticles containing different concentrations (0, 0.11, 0.16 0.22, 0.27 and 0.33 wt% in oil phase) of quercetin (Q_0.044_IO@Fu, Q_0.104_IO@Fu, Q_0.185_IO@Fu, Q_0.175_IO@Fu, Q_0.192_IO@Fu). (**c**) Quantitative analysis of flow cytometry results showing the percentage of apoptotic 4T1 BRCA-mutated cells treated with different quercetin concentrations. All data are expressed as mean ± standard deviation (SD) of at least three biologically different samples, unless otherwise mentioned.

Since quercetin serves as the PARP inhibitor in this system, its loading content within the QIO@Fu shell directly determines therapeutic efficacy. To identify the maximal effective payload, we evaluated the encapsulation efficiency across different concentrations of quercetin. Using the Folin–Ciocalteu colorimetric method, the encapsulated quercetin content was quantified for varying quercetin concentrations in the oil phase. As shown in Figure 2a, the quercetin loading reached a saturation plateau beyond 0.22 wt%, indicating the maximum capacity of the shell. To determine whether higher quercetin loading in QIO@Fu enhances apoptosis, we examined the cellular response of BRCA-mutated cells treated with the nanocarrier containing varying quercetin payloads. Figure 2b,c demonstrate that increasing quercetin loading resulted in a dose-dependent increase in the population of apoptotic cells. Based on these results, Q_0.185_IO@Fu (the QIO@Fu nanoparticle containing 0.185 mg quercetin) was selected as the optimal formulation. This composition represents the point of maximal effective loading before saturation, ensuring a sufficient therapeutic payload to induce significant apoptosis in BRCA-mutated cells.

**Figure 3:**
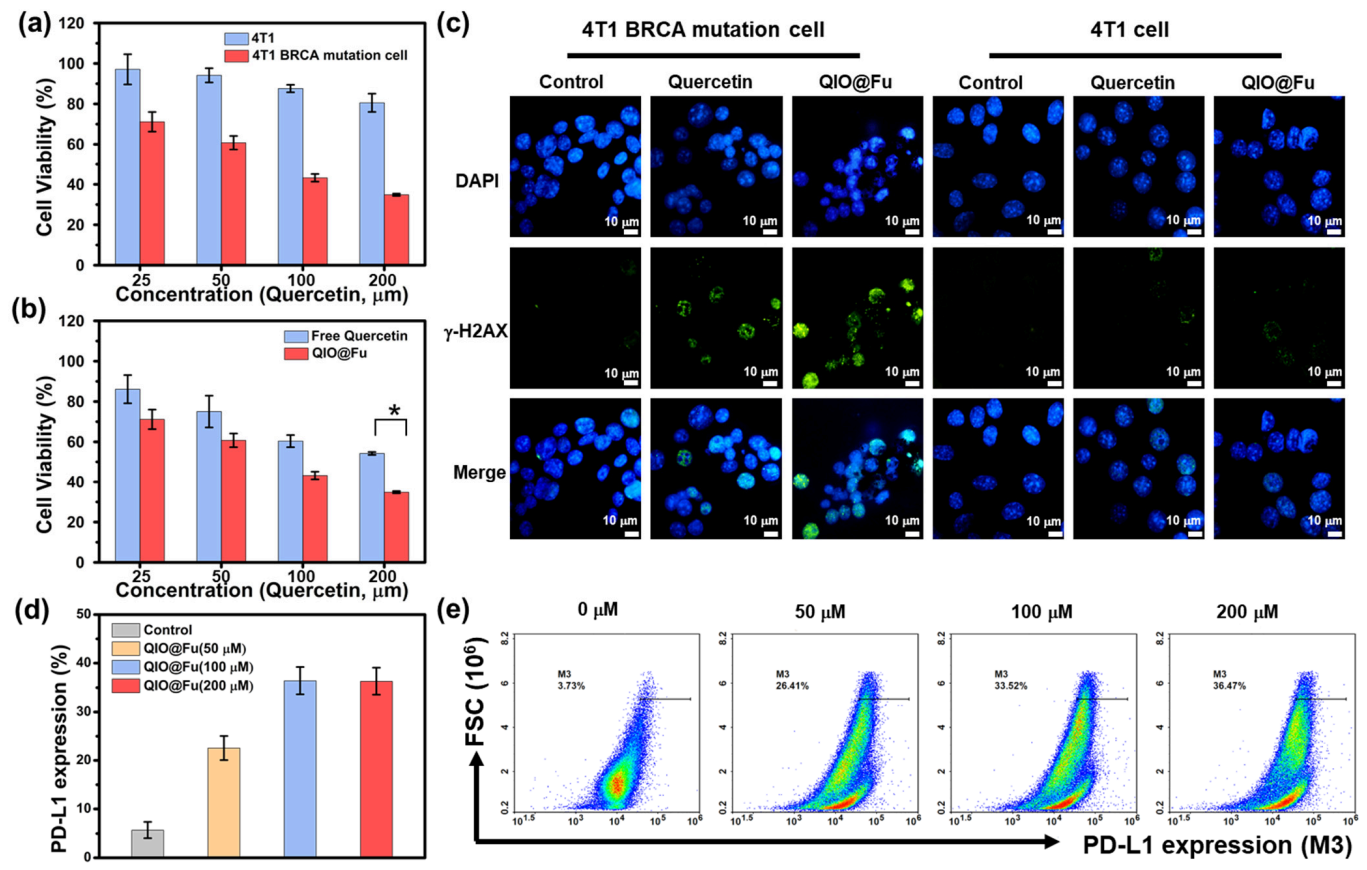
(**a**) Cell viability of 4T1and 4T1 BRCA-mutated cells treated with QIO@Fu nanoparticles. (**b**) Cell viability of 4T1 BRCA-mutated cells treated with quercetin and QIO@Fu nanoparticles. Two-tailed Student’s *t*-test; * *p* < 0.05. (**c**) Immunostaining of 4T1 BRCA-mutated cells after treatment with control, quercetin, and QIO@Fu. (Scale bar = 10 μm) (**d**) Quantification of PD-L1-positive cells (%) based on flow cytometry. (**e**) Flow cytometric analysis of cell surface PD-L1 expression in 4T1 cells after treatment with various concentrations of quercetin in QIO@Fu (0, 50, 100, and 200 μM). All data are expressed as mean ± standard deviation (SD) of at least three biologically independent samples, unless otherwise mentioned.

To evaluate the synthetic lethality of QIO@Fu in BRCA-mutant and non-mutant cells, we performed CCK-8 assays (Figure 3a). In non-mutated 4T1 cells, QIO@Fu maintained cell viability above 80% across all tested concentrations of QIO@Fu (25, 50, 100, and 200 µM quercetin equivalents), suggesting that the homologous recombination pathway effectively repairs DNA damage. In contrast, BRCA-mutant cells exhibited significant sensitivity to both free quercetin and QIO@Fu, demonstrating that the cell toxicity is selectively dependent on BRCA mutation. We then assessed whether QIO@Fu enhances this effect compared to the free drug in the sensitive BRCA-mutant 4T1 cell. As shown in Figure 3b, QIO@Fu decreased cell viability to 35% in 200 mM, whereas free quercetin plateaued at 50–60%. This plateau is attributed to the poor aqueous solubility of free quercetin, which limits its cellular uptake and intracellular accumulation [46,47,48]. These results confirm that QIO@Fu not only achieves selective cell toxicity towards BRCA-mutant 4T1 cells but also overcomes the delivery limitations of free quercetin to enhance therapeutic efficacy. We next examined whether the synthetic lethality of QIO@Fu induces substantial DNA double-strand breaks (DSBs). To validate DSB formation, γH2AX foci staining was conducted in BRCA-mutant and non-mutant cells [49,50]. This staining method identifies histone H2AX, which becomes gamma-phosphorylated at serine 139 (γH2AX) in response to DSB occurrence. The γH2AX staining in Figure 3c shows that no significant DNA damage was observed in non-mutated 4T1 cells treated with free quercetin and QIO@Fu, indicating low off-target effects. Conversely, BRCA-mutant 4T1 cells showed substantial γH2AX expression upon treatment with free quercetin and QIO@Fu. These data confirm that quercetin selectively induces DNA damage in BRCA-mutant cells via PARP inhibition. However, a potential trade-off exists. While quercetin inhibits PARP to induce DNA damage and apoptosis in BRCA-mutant cells, PARP inhibitors have been reported to upregulate PD-L1 by inactivating GSK3β, potentially facilitating immune evasion [10]. We observed that QIO@Fu treatment increased the percentage of PD-L1^+^ cell expression among BRCA-mutant cells (Figure 3d,e). This effect was more pronounced at higher concentrations of QIO@Fu (100 and 200 µM quercetin equivalents). Although the treatment effectively causes DNA damage and apoptosis, it simultaneously stimulates PD-L1 expression, which may limit the overall therapeutic durability.

### 3.2. Sequential Gene Delivery and PD-L1 Silencing of shPD-L1@QIO@Fu Nanomedicine

**Figure 4:**
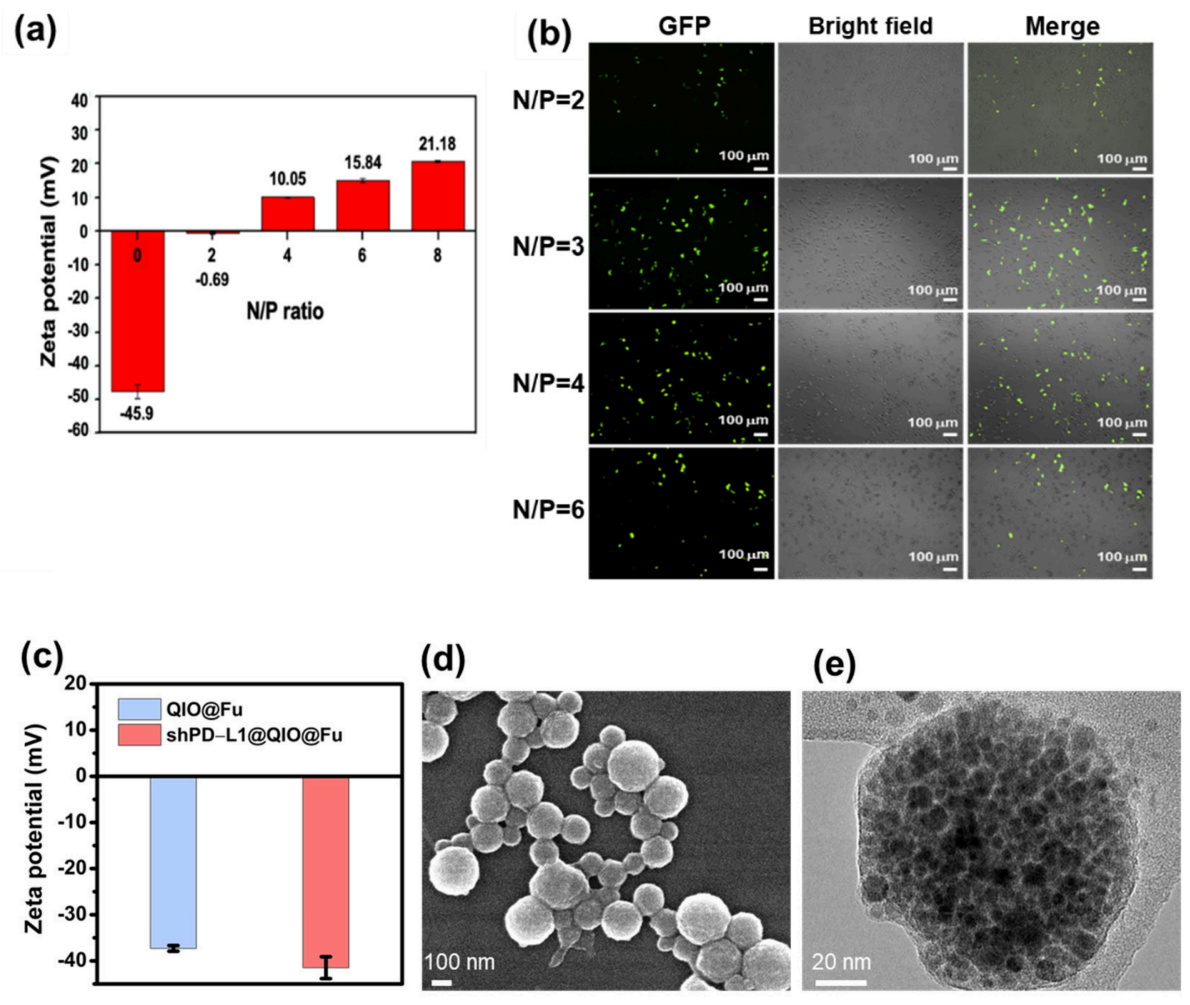
(**a**) Zeta potentials of PEI-DNA with different N/P ratios. (**b**) Cell transfection efficiency of PEI-DNA with different N/P ratios was observed by fluorescence microscope in 48 h. (Scale bar: 100 μm). (**c**) Zeta potential images of QIO@Fu and shPD-L1@QIO@Fu. (**d**) SEM images of shPD-L1@QIO@Fu. Scale bars: 100 nm. (**e**) TEM images of shPD-L1@QIO@Fu. Scale bars: 20 nm. All data are expressed as mean ± standard deviation (SD) of at least three biologically independent samples, unless otherwise mentioned.

To suppress PARP inhibition-induced PD-L1 upregulation, we employed a plasmid encoding short hairpin RNA targeting PD-L1 (shPD-L1) to silence its expression. To inhibit the PD-L1 protein expression, we evaluated the optimal transfection N/P ratio of polyethyleneimine (PEI) to shPD-L1 and the binding ability of the positive polymer PEI with the plasmid. Zeta potential analysis (Figure 4a) showed a surface charge reversal from negative to positive occurred at N/P = 2, indicating effective PEI-mediated plasmid binding. As shown in Figure 4b, a significant increase in fluorescence intensity of GFP expression was observed at N/P = 3, which maintained a healthy cell morphology. In contrast, at N/P ratios above 4, bright-field images revealed a progressive reduction in cell density and altered cell morphology indicative of the PEI-induced cytotoxicity, despite the presence of GFP signals. Thus, N/P = 3 was chosen for subsequent experiments, as it provided the optimal balance between gene delivery and cell viability. The shPD-L1@QIO@Fu nanomedicine was then fabricated by incorporating PEI/shPD-L1 polyplexes (N/P = 3) into the hydrophilic core of QIO@Fu via an emulsion-based method. Zeta potential measurements (Figure 4c) revealed that shPD-L1@QIO@Fu (−40.58 ± 2.36 mV) displayed a surface charge comparable to QIO@Fu (−38.16 ± 0.62 mV), suggesting that the PEI/shPD-L1 polyplexes were efficiently encapsulated within the inner layer rather than adsorbed on the surface. The quercetin content was 0.181 ± 0.022 mg, showing no significant difference from QIO@Fu. Furthermore, DNA loading efficiency assessed using GelRed dye was 92.65%, confirming successful complexation and encapsulation. Scanning electron microscopy (SEM) in Figure 4d confirmed that shPD-L1@QIO@Fu retained the structure and similar particle size to QIO@Fu, indicating that nanoparticle integrity was preserved after shPD-L1 loading. The average particle diameter of shPD-L1@QIO@Fu measured by dynamic light scattering (DLS) was about 255.7 ± 60.3 nm in aqueous solution (Figure S1). The transmission electron microscopy (TEM) image in Figure 4e further confirms that shPD-L1-loaded QIO@Fu formed a solid particle structure.

**Figure 5:**
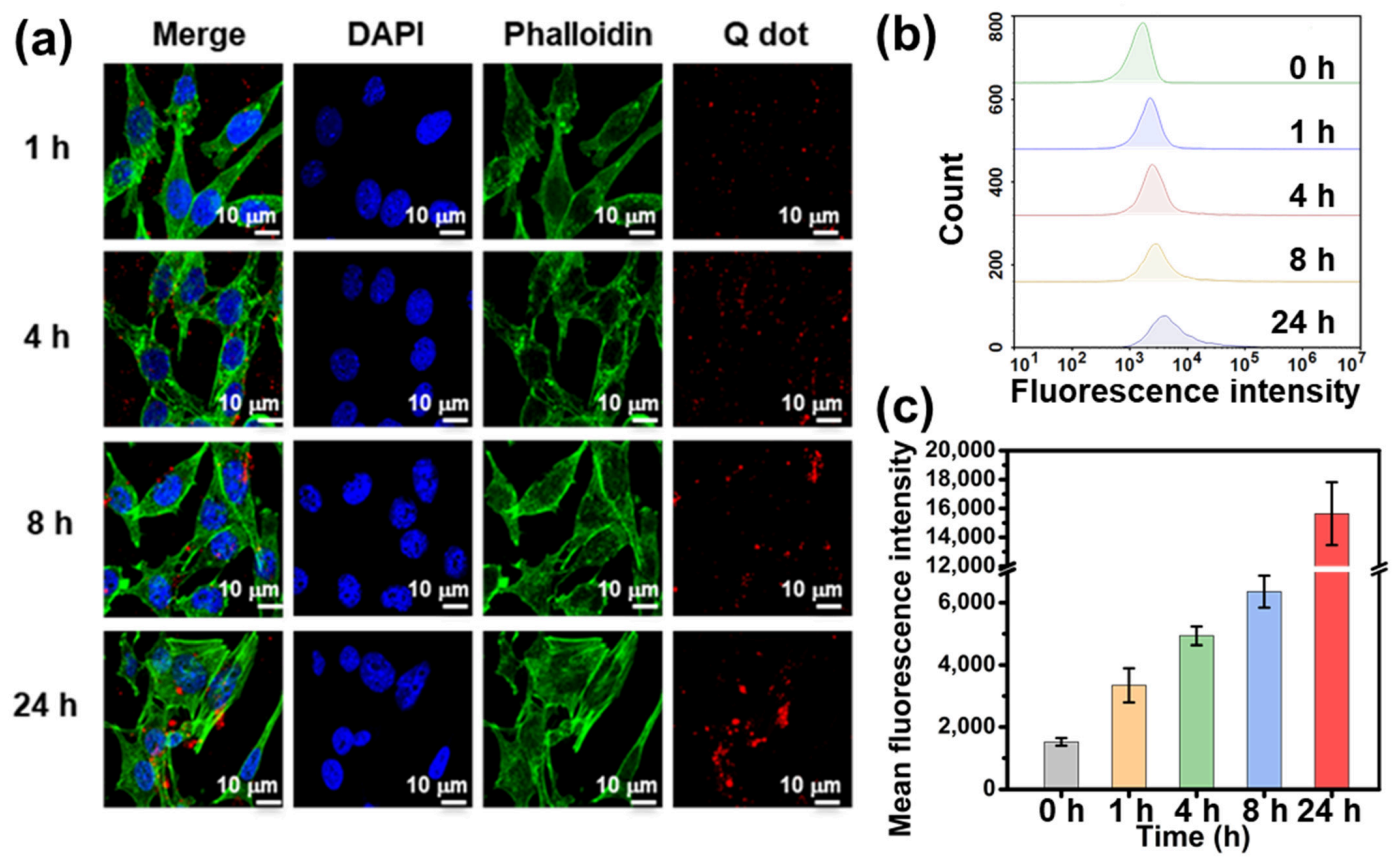
(**a**) Confocal fluorescence images showing cellular uptake and distribution of shPD-L1@QIO@Fu in 4T1 cells after 8 h and 24 h incubation. Blue: nuclei (DAPI); green: cytoskeleton (Phalloidin); red: Q dot-labeled nanoparticles. (**b**) Fluorescence intensity of Qd-shPD-L1@QIO@Fu by flow cytometry at 0, 1 h, 4 h, 8 h and 24 h. (**c**) Histogram of mean fluorescence intensity of shPD-L1@QIO@Fu at 1 h, 4 h, 8 h and 24 h by flow cytometry, presented as mean ± standard deviation (SD) from *n* = 3 independent experiments.

We next monitored the intracellular trafficking and accumulation of shPD-L1@QIO@Fu in BRCA-mutant 4T1 cells. Confocal microscopy and flow cytometry (Figure 5a–c) revealed the intracellular uptake of shPD-L1@QIO@Fu. At 1 h post-incubation, only weak red fluorescence signals were observed and scattered at the cell periphery. From 4 h to 8 h, the intracellular red fluorescence gradually increased, indicating substantial internalization of shPD-L1@QIO@Fu into the cytoplasm. By 24 h, intense fluorescence signals were detected, showing extensive accumulation of the shPD-L1@QIO@Fu, which appeared partially aggregated and clustered in the perinuclear region.

**Figure 6:**
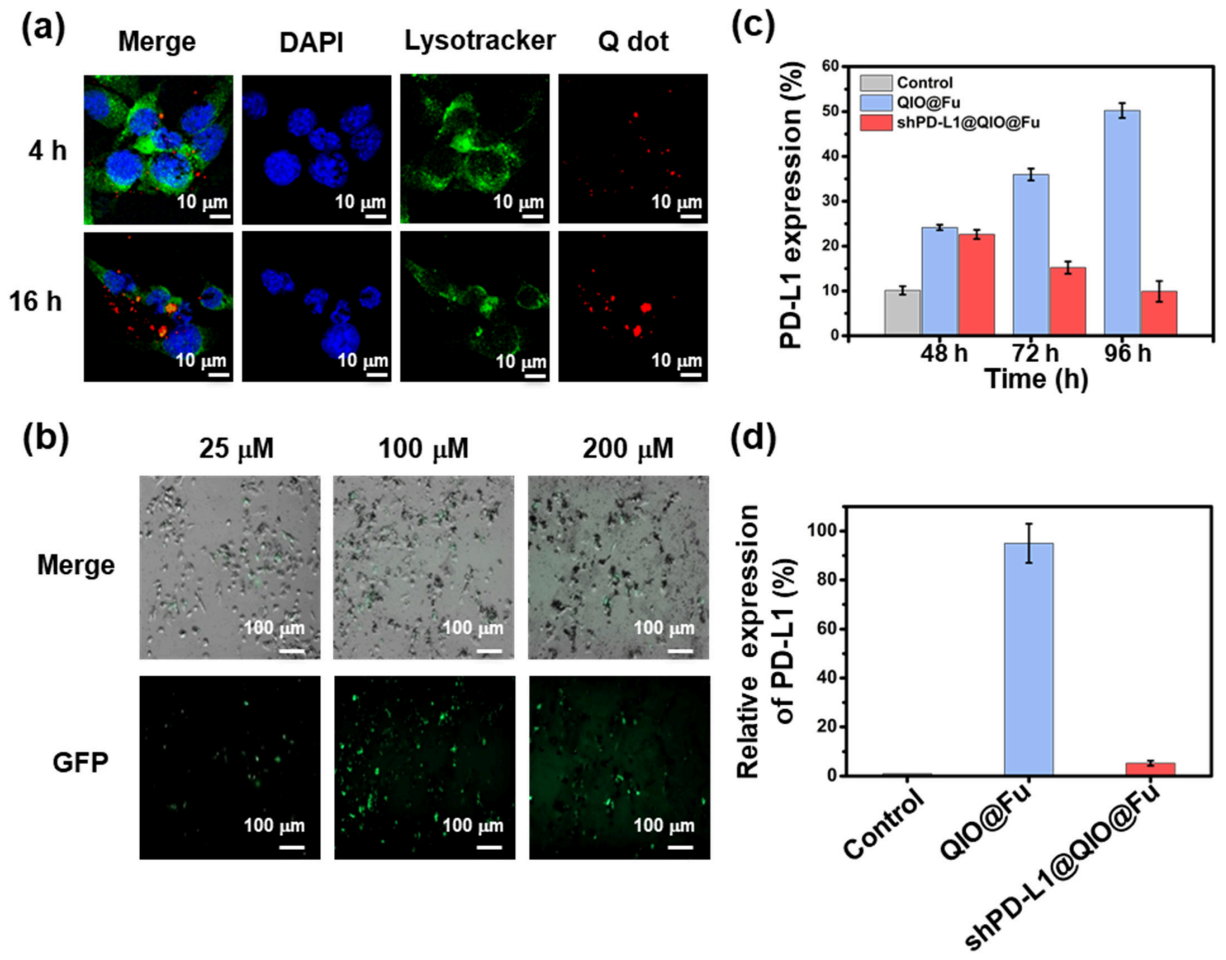
(**a**) Intracellular colocalization analysis of shPD-L1@QIO@Fu and lysosomes in 4T1 cells after 4 h and 16 h incubation. Blue: nuclei; green: lysosomes (LysoTracker); red: Q dot-labeled nanoparticles. (**b**) Time-dependent PD-L1 expression on the cell surface of 4T1 cells treated with QIO@Fu or shPD-L1@QIO@Fu measured at 48, 72, and 96 h. (*n* = 3) (**c**) GFP protein expression in 4T1 cells at 48 h post-transfection with shPD-L1@QIO@Fu, observed by fluorescence microscopy. (**d**) Relative *PD-L1* mRNA expression (normalized to *GAPDH*) in 4T1 BRCA-mutant cells treated with QIO@Fu and shPD-L1@QIO@Fu for 72 h. All data are expressed as mean ± standard deviation (SD) of at least three biologically independent samples, unless otherwise mentioned.

To evaluate endosomal escape, we examined the intermediate stage at 4 h and 16 h using Lysotracker staining (Figure 6a). Some nanoparticles showed colocalization with lysosomes (yellow), confirming endocytic uptake. However, the observation of red fluorescence signals dissociated from green lysosomal staining indicated successful endosomal escape, a process attributed to the proton sponge effect of PEI triggered by the acidic endosomal environment. Since PARP inhibition-induced synthetic lethality initiates PD-L1 transcription within 24–48 h, we anticipated a subsequent increase in cell surface PD-L1 expression. Notably, nuclear gene delivery was already evident by 48 h as indicated by GFP fluorescence (Figure 6c), confirming transfection across all tested concentrations of shPD-L1@QIO@Fu (25, 100, and 200 µM quercetin equivalents). Quantitative analysis of GFP-positive cells showed that the transfection efficiency reached 33% at 100 µM. When the concentration was increased to 200 µM, the efficiency decreased to 26%, possibly due to cytotoxicity at high quercetin concentrations. Then, we performed quantitative analysis using flow cytometry. As shown in Figure S2a, the fluorescence intensity of cells treated with shPD-L1@QIO@Fu showed a significant rightward shift compared to the control and QIO@Fu groups, indicating effective cellular uptake and successful gene delivery. Further quantitative evaluation of the mean fluorescence intensity (MFI) as shown in Figure S2b confirmed a significantly higher MFI for the shPD-L1@QIO@Fu group. Additionally, we quantified the cell transfection rate. The shPD-L1@QIO@Fu group exhibited an increase in cell transfection efficiency, achieving approximately 31% (Figure S2c). These findings provide quantitative evidence of the highly efficient gene transfection of shPD-L1@QIO@Fu. To capture the dynamics of cell surface PD-L1 regulation, we monitored the percentage of PD-L1^+^ cells at 48, 72, and 96 h using flow cytometry (Figure 6c). QIO@Fu and shPD-L1@QIO@Fu exhibited an increased percentage of PD-L1^+^ cells at 48 h, indicating an initial DNA damage-driven upregulation of PD-L1. However, only shPD-L1@QIO@Fu exhibited a time-dependent decrease in PD-L1^+^ cells from 72 h to 96 h, suggesting that nuclear delivery of shPD-L1 and subsequent inhibition at the mRNA level likely occurred between 48 and 72 h. Furthermore, *PD-L1* mRNA levels in the shPD-L1@QIO@Fu group were reduced to approximately 5% of the control at 72 h (Figure 6d), effectively reversing the quercetin-induced upregulation. These results demonstrate a dual therapeutic effect: quercetin induces DNA damage and tumor cell death, while the co-delivered shPD-L1 counteracts the compensatory PD-L1 upregulation, thereby preventing potential immune evasion. To further evaluate the therapeutic effect at the cellular level, we conducted an apoptosis assay. The apoptosis assay was performed using an equivalent quercetin concentration of 200 µM, which was determined based on cell viability. As shown in S2, the apoptosis rates were 9.34 ± 0.596%, 37.5 ± 0.655%, and 57.93 ± 2.29% for the control, quercetin alone, and shPD-L1@QIO@Fu groups, respectively, thereby confirming the significant apoptotic effect of shPD-L1@QIO@Fu.

**Figure 7:**
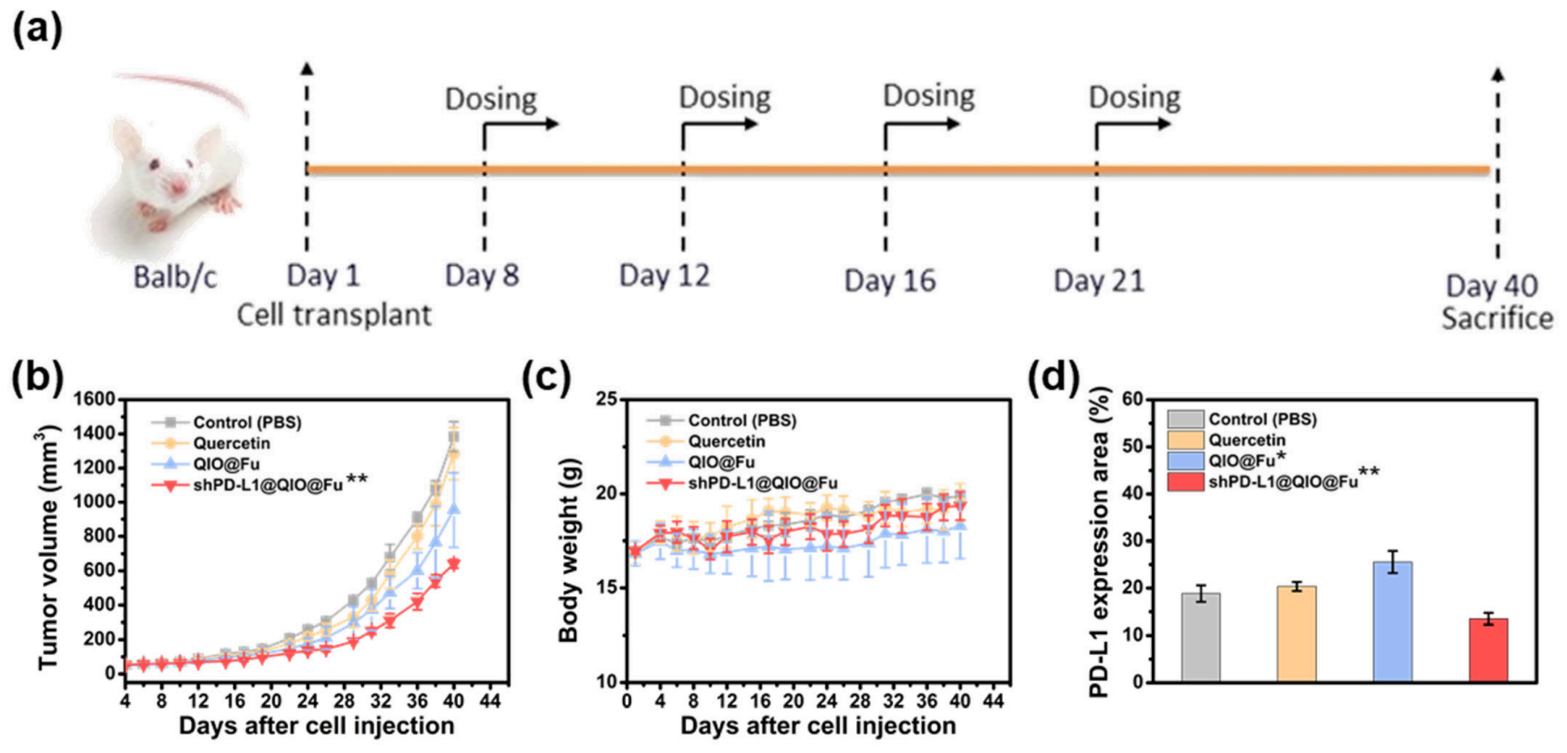
(**a**) Schematic timeline of the animal experiment. BALB/c mice were inoculated with tumor cells on Day 1, followed by drug administration on Days 8, 12, 16, and 21. Mice were sacrificed for analysis on Day 40. (**b**) Tumor volume growth curves over time in four treatment groups: control (PBS), quercetin, QIO@Fu, and shPD-L1@QIO@Fu. Statistical analysis was performed using one-way ANOVA with the Tukey’s multiple comparisons followed by a post hoc test. (**c**) Changes in mouse body weight during treatment in each group. (**d**) Analysis of PD-L1 protein expression in tumor tissues from each group. Tumor samples were collected and subjected to immunohistochemical (IHC) staining to assess PD-L1 protein levels, two-tailed Student’s *t*-test. * *p* < 0.05 and ** *p* < 0.01.

To evaluate the in vivo therapeutic efficacy of shPD-L1@QIO@Fu, we established a BRCA-mutated 4T1 breast cancer mouse model. As illustrated in Figure 7a, tumor-bearing mice were constructed by cell inoculation on Day 1. Treatments were initiated on Day 8 and subsequently administered intraperitoneally on Days 12, 16, and 21, followed by animal sacrifice and tissue collection on Day 40. Mice were randomized into four groups: control (PBS), quercetin, QIO@Fu, and shPD-L1@QIO@Fu. As shown in Figure 7b, both the control and free quercetin groups exhibited rapid and comparable tumor growth. This limited efficacy of free quercetin is likely attributed to its poor bioavailability and rapid metabolism in vivo, preventing the accumulation of therapeutic concentrations required for cytotoxicity. In contrast, the QIO@Fu group showed only a moderate inhibition of tumor growth, while shPD-L1@QIO@Fu achieved a substantially greater reduction in tumor volume. Furthermore, no significant bodily weight loss was observed in any group (Figure 7c), indicating favorable safety across all treatments. To validate the in vivo gene silencing efficiency, we performed immune histochemical (IHC) analysis of the tumor tissues (Figure 7d and Figure S4). Tumors treated with the QIO@Fu groups exhibited upregulated PD-L1 expression compared to controls, suggesting that this upregulation was induced by quercetin-mediated DNA damage. Conversely, shPD-L1@QIO@Fu resulted in a reduction of PD-L1 levels in tumor regions, confirming effective gene silencing in vivo. These results demonstrated that co-delivery of quercetin with shPD-L1 can suppress tumor growth in BRCA-mutant models by simultaneously inducing DNA damage and modulating the PD-L1 adaptive response.

**Figure 8:**
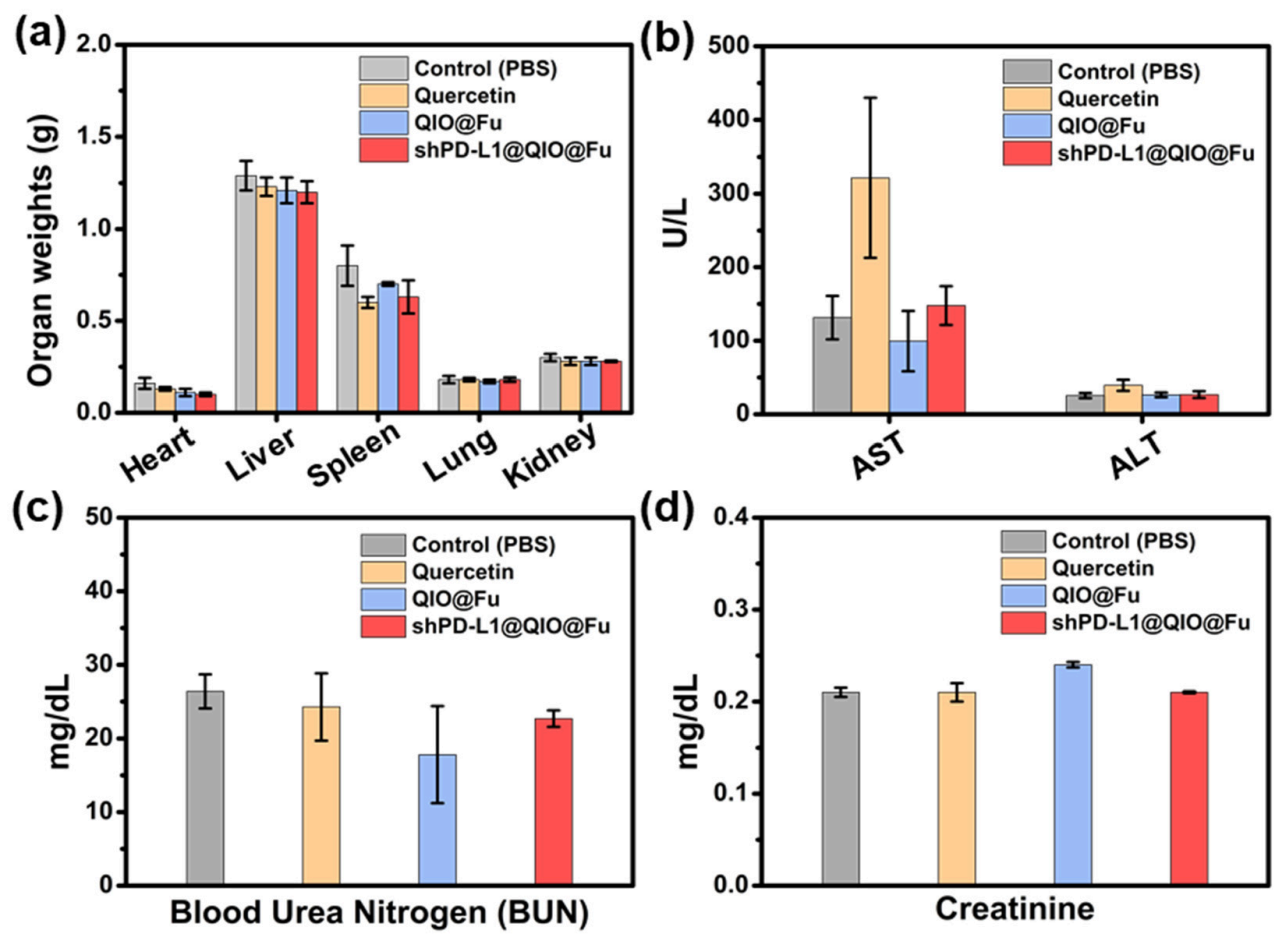
(**a**) Organ weights of major mouse organs following different treatments (control, quercetin, QIO@Fu and shPD-L1@QIO@Fu). (**b**) Serum aspartate aminotransferase (AST) and alanine aminotransferase (ALT) levels reflecting liver function following different treatments (control, quercetin, QIO@Fu and shPD-L1@QIO@Fu). (**c**) Blood urea nitrogen (BUN) levels for kidney function following different treatments (control, quercetin, QIO@Fu and shPD-L1@QIO@Fu). (**d**) Serum creatinine (CRE) levels indicating kidney function following different treatments (control, quercetin, QIO@Fu and shPD-L1@QIO@Fu); all groups maintained within the normal physiological interval. Data are presented as mean ± SD (*n* = 6).

Safety evaluation is a critical aspect in the development of shPD-L1@QIO@Fu in our BRCA-mutated breast cancer mouse model. Throughout the experimental period, daily clinical observations and weekly monitoring of body weight revealed no animal mortality or significant adverse clinical signs across all treatment groups. At the study endpoint (Day 40), gross anatomical dissection of major organs, including the heart, liver, spleen, lungs, and kidneys, showed no evidence of visible organ lesions in any group. Organ weights also remained consistent across treatments (Figure 8a), further indicating minimal systemic toxicity. To evaluate potential toxicity, serum biochemical analyses of liver and kidney function were tested in Figure 8b and Figure S5. As compared to the control, aspartate aminotransferase (AST) and alkaline phosphatase (ALP) levels were elevated in the quercetin group. However, alanine aminotransferase (ALT), albumin (ALB) and gamma-glutamyl transferase (GGT), were not elevated, indicating mild hepatic effects. In contrast, QIO@Fu, and shPD-L1@QIO@Fu maintained these markers at normal levels, suggesting that nanocarrier encapsulation effectively mitigates the hepatic side effects associated with free quercetin. Assessment of renal function using blood urea nitrogen (BUN) and creatinine (CRE) (Figure 8c,d) demonstrated no statistically significant differences among the control, quercetin, QIO@Fu, and shPD-L1@QIO@Fu groups. The absence of adverse effects in organ weight and serum biochemical markers supports the translational potential of shPD-L1@QIO@Fu for safe and effective cancer combination therapy.

## 4. Limitations and Outlook

While this work establishes a proof of concept for the shPD-L1@QIO@Fu nanomedicine in synergistic chemo/gene therapy, several considerations remain for future development. Several aspects warrant further investigation to advance this platform toward translational application, particularly with respect to achieving more comprehensive immune escape blockade (e.g., CD8^+^ T-cell infiltration, IFN-γ/TNF-α secretion, and other inflammatory cytokine levels or T cell-mediated tumor cell killing). Moreover, in vivo evidence of immune activation to directly link PD-L1 silencing with enhanced antitumor immunity need further study. Additionally, a biosafety profile would ideally include assessments such as hemolysis assays and erythrocyte aggregation assays, which were not performed in this study but are critical for future translational applications. Furthermore, pharmacokinetics and biodistribution are essential to characterize the in vivo behavior and targeting efficacy of shPD-L1@QIO@Fu, and these will be critical components of future research. Nevertheless, these findings demonstrate its efficient gene transfection capability and establishing a proof of concept for the synergistic antitumor effects arising from PARP inhibitor-induced synthetic lethality combined with PD-L1 gene silencing.

## 5. Conclusions

In summary, our study establishes a multifunctional nanomedicine that enables effective quercetin loading and co-encapsulation of hydrophilic shPD-L1 DNA via a double emulsion process. By combining PARP inhibition-induced synthetic lethality with targeted gene silencing, shPD-L1@QIO@Fu exerts a dual therapeutic effect against BRCA-mutant tumor cells by inducing DNA double-strand breaks and downregulating PD-L1 expression. These findings present a potential therapeutic strategy for the treatment of BRCA-mutant cancers.

## Figures and Tables

**Table 1:** Polymerase chain reaction (PCR) conditions.

Step	Temperature	Time	Cycle
Initial denaturation	95 °C	15 min	1×
Denaturation	95 °C	10 s	40×
Annealing/extension	95 °C	30 s	40×
Complete extension	95 °C	10 s	1×
Melting curve detection	65–95 °C	5 s per 0.5 °C	1×

## Data Availability

The authors declare that the data supporting the findings of this study are available in the article, source data, and Supplementary Information. A summary of this article is provided in Supplementary Information. The source data are provided in this study. Additional relevant data are available from the corresponding author upon request.

## Supplementary Materials

The following supporting information can be downloaded at: https://www.mdpi.com/article/10.3390/polym18111314/s1, Figure S1: Zeta potentials of PEI-DNA with different N/P ratio; Figure S2: (a) Flow cytometry histograms of the fluorescence intensity in cells treated with control, QIO@Fu, and shPD-L1@QIO@Fu. (b) Quantitative analysis of mean fluorescence intensity (MFI) derived from the flow cytometry data for the control, QIO@Fu, and shPD-L1@QIO@Fu groups. (c) Quantification of cell transfection efficiency, expressed as the percentage of fluorescent cells, after treatment with control, QIO@Fu, and shPD-L1@QIO@Fu. Data are presented as mean ± standard deviation (n = 3), two-tailed Student’s *t*-test; * *p* < 0.05 and ** *p* < 0.01; Figure S3: (a) Apoptosis of 4T1 BRCA-mutated cells after incubation with quercetin and shPD-L1@QIO@Fu (200 µM, quercetin) in flow cytometry (c) Quantitative analysis of flow cytometry results showing the percentage apoptotic 4T1 BRCA-mutated cells treated with quercetin and shPD-L1@QIO@Fu (200 µM, quercetin). n = 3, two-tailed Student’s *t*-test; * *p* < 0.05, ** *p* < 0.01, and *** *p* < 0.001; Figure S4: Gross view of anti-PD-L1 immunohistochemical staining in 4T1-BRCA mutant cancer tumor samples following different treatments (control, quercetin, QIO@Fu, and shPD-L1@QIO@Fu). Scale bar: 0.25 cm; Figure S5: Serum albumin (ALB), alkaline phosphatase (ALP), and gamma-glutamyl transferase (GGT) levels reflecting liver function following different treatments (control, quercetin, QIO@Fu, and shPD-L1@QIO@Fu).
