# Supplementary material for: Synthesis and Characterization of Dual Natural Quercetin/Fucoidan Gene Delivery Nanoplatform for Synthetic Lethality in BRCA-Deficient Tumors
Source: Polymers (Basel). 2026 May 26;18(11):1314. doi: 10.3390/polym18111314 (PMC13259162; doi:10.3390/polym18111314)
Supplement: Supplementary file 1 [file polymers-18-01314-s001.zip › polymers-4284928-supplementary.pdf]

Supporting Information

Synthesis and Characterization of Dual Natural Quercetin/Fucoidan Gene Delivery Nanoplatform for Synthetic Lethality in BRCA-Deficient Tumors

Jih-Hao Yeh [1,†], Shih-Yu Huang [1,†], Ching-Chun Chu [1], Chun-Tao Su [1], Hung-Wei Cheng [2,*] and San-Yuan Chen [1,3,4,*]

1 Department of Materials Science and Engineering, National Yang Ming Chiao Tung University, Hsinchu 300093, Taiwan; aasd3431@gmail.com (J.-H.Y.); e881122e881122@gmail.com (S.-Y.H.); seaftseaft@gmail.com (C.-C.C.); chuntaosu.en09@nycu.edu.tw (C.-T.S.)
2 Department of Biological Science and Technology, China Medical University, Taichung 406040, Taiwan
3 Graduate Institute of Biomedical Science, China Medical University, Taichung 406040, Taiwan
4 School of Dentistry, College of Dental Medicine, Kaohsiung Medical University, Kaohsiung 807378, Taiwan
* Correspondence: chenghw@cmu.edu.tw (H.-W.C.); sanyuanchen@nycu.edu.tw (S.-Y.C.)
† These authors contributed equally to this work.

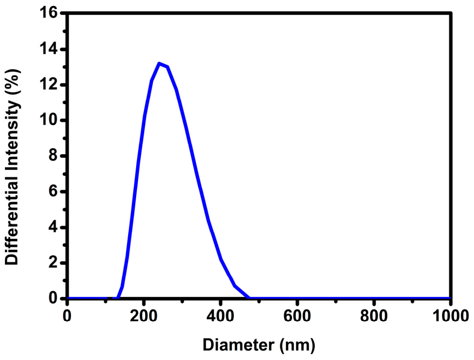

Figure S1. Zeta potentials of PEI-DNA with different N/P ratio.

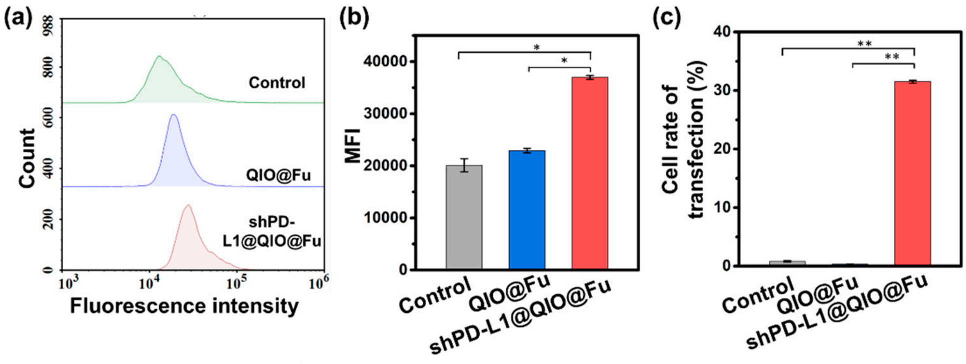

Figure S2. (a) Flow cytometry histograms of the fluorescence intensity in cells treated with control, QIO@Fu, and shPD-L1@QIO@Fu. (b) Quantitative analysis of mean fluorescence intensity (MFI) derived from the flow cytometry data for the control, QIO@Fu, and shPD-L1@QIO@Fu groups. (c) Quantification of cell transfection efficiency, expressed as the percentage of fluorescent cells, after treatment with control, QIO@Fu, and shPD-L1@QIO@Fu. Data are presented as mean ± standard deviation (n=3), two-tailed Student's t-test; *$p < 0.05$ and **$p < 0.01$.

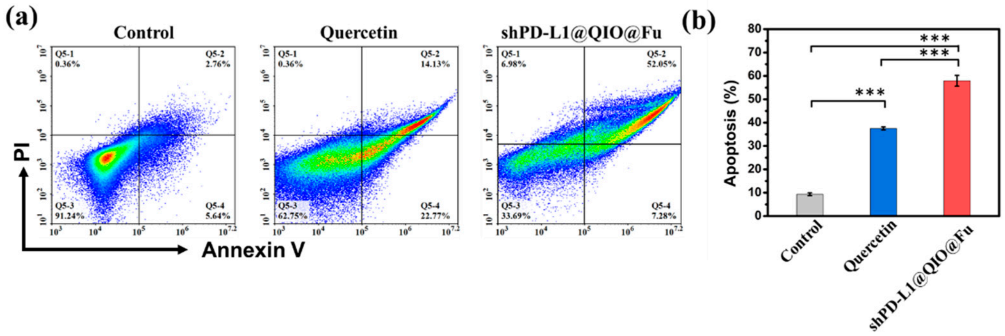

Figure S3. (a) Apoptosis of 4T1 BRCA-mutated cells after incubation with Quercetin and shPD-L1@QIO@Fu (200 μM, quercetin) in Flow cytometry (c) Quantitative analysis of flow cytometry results showing the percentage apoptotic 4T1 BRCA-mutated cells treated with Quercetin and shPD-L1@QIO@Fu (200 μM, quercetin). n = 3, two-tailed Student's t-test; $*p < 0.05$, $**p < 0.01$, and $***p < 0.001$.

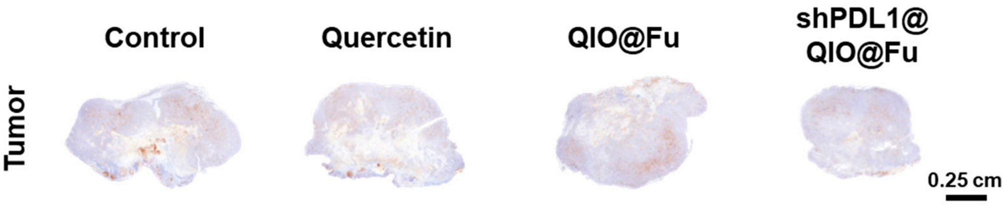

Figure S4. Gross view of anti-PD-L1 immunohistochemical staining in 4T1-BRCA mutant cancer tumor samples following different treatments (control, Quercetin, QIO@Fu, and shPD-L1@QIO@Fu). Scale bar: 0.25 cm.

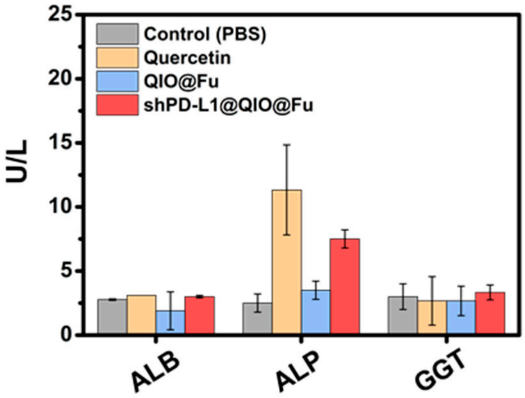

Figure S5. Serum albumin (ALB), alkaline phosphatase (ALP), and gamma-glutamyl transferase (GGT) levels reflecting liver function following different treatments (control, Quercetin, QIO@Fu, and shPD-L1@QIO@Fu).
